# Dr. Benjamin Travers

**Published:** 1858-05

**Authors:** 


THE LATE MR. TRAVERS.—Our London Exchanges bring us the news of the death of Benjamin Travers, Esq., F.R.S., for many years one of the most active and distinguished surgeons of Great Britain. It took place on the 6th of March, in the seventy-fifth year of his age, having been in the practice of his profession for upward of half a century. Mr. Travers was a pupil of the late Sir Astley Cooper, and at the time of his death was Serjeant-Surgeon to her Majesty Queen Victoria, having been appointed to this office only a few weeks before his demise. He was the author of a number of works, the first of which was published while he was Demonstrator of Anatomy at Guy's Hospital in 1812, under the title of *An Inquiry into the Process of Nature in Repairing Wounds of the Intestines.* This work gave him at once a high position in the profession, and was, for a long time, the only treatise on the subject in the English language. Subsequently he published, along with Sir Astley Cooper, a volume of essays on various surgical subjects. In 1818 he issued a work upon diseases of the eye, founded mainly upon personal observation at the Royal London Ophthalmic Hospital, and which was soon after republished in this country, with notes by Dr. Delafield, of New York. His last literary production, we believe, was his work upon *Inflammation*, published nearly twenty years ago. This work, which was extensively circulated both in Europe and this country, had the effect of calling attention to a subject which had, until then, been too much neglected. He also contributed numerous papers to periodical medical literature, especially to the Transactions of the Medico-Chirurgical Society of London. In 1816 he was elected one of the Surgeons to St. Bartholomew's Hospital, a situation which he retained for many years. Mr. Travers has left a worthy representative in his son, Benjamin Travers, Esq., Jr., already a man of professional distinction.

